# Pyrogenic carbon production from fires in China from 1901 to 2020

**DOI:** 10.1016/j.isci.2026.116060

**Published:** 2026-05-22

**Authors:** Chenyi Yuan, Mengjie Han, Minxuan Sun, Bo Pan, Qing Zhao, Wei Li

**Affiliations:** 1 Ministry of Education Key Laboratory of Groundwater Circulation and Environmental Evolution, China University of Geosciences (Beijing), Beijing 100083, China; 2 Department of Earth System Science, Ministry of Education Key Laboratory for Earth System Modeling, Institute for Global Change Studies, Tsinghua University, Beijing 100084, China; 3 Faculty of Environmental Science & Engineering, Kunming University of Science & Technology, Kunming 650500, China; 4 Guangdong Key Laboratory of Integrated Agro-environmental Pollution Control and Management, Institute of Eco-environmental and Soil Sciences, Guangdong Academy of Sciences, Guangzhou 510650, China

**Keywords:** Atmospheric chemistry, Atmospheric science, Forestry, Global carbon cycle

## Abstract

Fires are a major disturbance in the terrestrial ecosystem carbon cycle, releasing CO_2_ while converting part of biomass into pyrogenic carbon (PyC). PyC is increasingly recognized as a persistent carbon pool, yet it remains largely overlooked in the global carbon budget, and its large-scale and long-term dynamics remain poorly quantified. We quantified the spatiotemporal patterns of PyC production in China from 1901 to 2020 by integrating newly reconstructed century-scale burned area data and fuel-type-specific PyC production factors. Mean annual PyC production was 0.8 Tg C yr^−1^ for forests and 0.0059 Tg C yr^−1^ for non-forest fires, corresponding to 17.8% and 12.3% of associated CO_2_ emissions. PyC production increased from 1901 to 2004 and declined thereafter, with Northeast China dominating national totals. This study provides the first century-long assessment of PyC dynamics in China and delivers gridded, uncertainty-quantified PyC estimates that can be incorporated into fire CO_2_ inventories and carbon-accounting assessments.

## Highlights

• PyC production peaked around 2004 then declined in forest and non-forest fires
• PyC equals 12%–18% of fire CO_2_ emissions across ecosystems (1901–2020)
• Northeast dominated PyC over the century; North led non-forest PyC recently

## Graphical abstract

**Figure:**
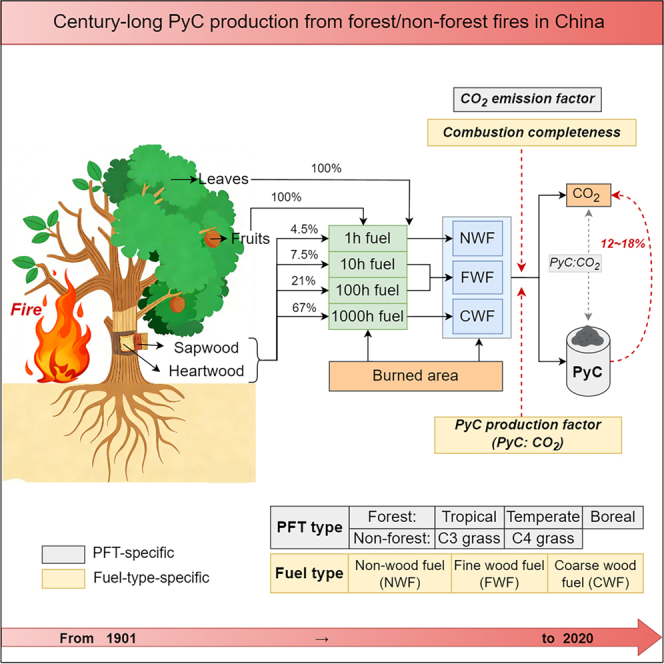

## Introduction

Fires are among the most important disturbances in terrestrial ecosystems, releasing carbon that has been stored in vegetation for decades to centuries into the atmosphere over short timescales. In natural landscapes, fires commonly occur across both forest and non-forest biomes, many of which exhibit high fire risk, as evidenced by the extreme forest fires in Canada^1^ and the frequent savanna and grassland fires in Africa.^2^ In addition to emitting large quantities of greenhouse gases and aerosols, fires generate pyrogenic carbon (PyC) through the incomplete combustion of biomass.^3^^,^^4^ Because of its highly condensed molecular structure, PyC decomposes far more slowly than uncharred organic matter and can persist in soils, sediments, and aquatic systems for centuries to millennia.^5^^,^^6^^,^^7^ This slow turnover makes PyC a potentially important long-term carbon pool that reduces the immediate release of fire-derived carbon to the atmosphere. However, the production and spatiotemporal dynamics of PyC remain poorly quantified and are largely overlooked in current global carbon budgets.^8^^,^^9^^,^^10^ Under future warming, climate-induced risks, such as wildfires and extreme drought are expected to intensify, with global CO_2_ emissions from forest fires increasing by approximately 1% per year,^11^ thereby further weakening the vegetation carbon sink.^12^ As a result, the role of PyC as a long-term carbon reservoir is expected to become increasingly important. Therefore, accurately estimating PyC production across ecosystems and characterizing its spatiotemporal variability are crucial for improving our understanding of its contribution to fire emissions and the global carbon cycle, as well as for advancing fire-related process representations in Earth system models. In this study, we focus specifically on China, where diverse climatic conditions, ecosystem types, and historical fire dynamics make it a critical region for long-term PyC assessments.

Among global regions, China represents an important yet under-constrained case, due to its long fire history, rapid land-use change, and limited availability of consistent long-term burned-area records. However, current estimates of PyC production at regional and global scales remain limited, as they largely rely on fire-exposure experiments and postfire field soil sampling in severely burned areas.^13^^,^^14^ It constrains the ability to quantify large-scale and long-term spatial patterns of PyC production. Because PyC is a direct product of biomass combustion, its generation is closely linked to fire-induced CO_2_ emissions. Recent studies have explored this relationship by defining PyC production factors as the ratio of PyC to CO_2_ (PyC:CO_2_),^9^^,^^15^ providing a practical means of estimating PyC production from fire emissions. Jones et al.^9^ compiled a comprehensive database of PyC production factors for four biomass components across 144 burn units and subsequently estimated global spatiotemporal PyC dynamics by applying the PyC:CO_2_ ratio to fire emissions data from the Global Fire Emissions Database (GFED4s).^16^ Their analysis yielded a mean estimate of global PyC production of 256 TgC yr^−1^ over 1997–2016.^9^ Bowring et al.^8^ incorporated the PyC formation process into the fire module of the dynamic vegetation model by explicitly linking fire-induced CO_2_ emissions to PyC production, enabling simulation of PyC formation and its contribution to the terrestrial carbon budget. Their results indicated an annual soil carbon accumulation from PyC of 337 TgC yr^−1^ during 1901–2010.^8^ However, PyC dynamics remain rarely presented in Earth system models due to the lack of long-term, spatially explicit burned-area observations for model validation and the inherent complexity of fire processes. Most dynamic vegetation models still struggle to accurately reproduce fire emissions and fail to capture the spatial patterns of PyC production in China,^8^^,^^17^ highlighting the urgent need for reliable burned area and PyC production datasets to improve representation of fire processes in modeling frameworks.

Because many large-scale PyC estimates are derived by applying PyC production factors to fire CO_2_ emissions, robust CO_2_ emission estimates are a prerequisite for quantifying PyC production. Fire CO_2_ emissions can be estimated using bottom-up, emission-factor (EF)-based inventories and top-down atmospheric inversions,^18^^,^^19^ in addition to fire-enabled ecosystem models. Owing to the complexity of fire processes, the emission factor method is widely applied for estimating fire CO_2_ emissions from regional to global scales, which integrates fuel load, burned area, combustion efficiency, and EFs.^16^^,^^20^^,^^21^ This bottom-up approach enables attribution of emissions to specific sources and locations, but its accuracy depends heavily on the quality of key input data, particularly burned area and biomass datasets. The burned area data are primarily provided by remote sensing in the satellite era. Fire emissions in China have been estimated for various periods and ecosystems using different methods, such as forest fire emissions during 1950–2000,^22^ 1988–2012,^23^ and 2000–2022.^18^ Additional estimates for multiple biomes—such as forest, savannas, peatlands, and agricultural ecosystems—are available from GFED4s for 1997–2016^16^ and for forest, shrubland, grassland, and cropland during 2003–2017.^20^ However, estimates from the pre-satellite era remain scarce and highly uncertain,^24^ thereby bringing uncertainties into assessments of PyC production. These limitations highlight the need to integrate long-term, high-precision burned-area datasets with a unified estimation approach to more reliably assess the long-term variability of PyC production in China.

In this study, we combined long-term forest and non-forest burned area data for 1901–2020 reconstructed from satellite data and machine learning^25^ with historical biomass data simulated using a state-of-the-art dynamic global vegetation model (ORCHIDEE) with explicit calibrations for China over the same period.^17^ Compared with existing global products, the observation-constrained burned area dataset provides a longer temporal coverage extending back to the pre-satellite era, while the biomass simulations benefit from China-specific calibrations that reduce biases associated with globally parameterized vegetation models. These improvements enable a more robust and regionally constrained assessment of long-term PyC production. By incorporating these datasets with additional parameters, such as combustion completeness, EFs, and PyC production factors (Figure S1), we estimated the spatiotemporal dynamics of PyC production from forest and non-forest (mainly natural grasslands, excluding managed croplands and pastures; see STAR Methods) fires in China over the past century. We further quantified the proportion of PyC production relative to fire-induced CO_2_ emissions. The resulting gridded, uncertainty-quantified PyC dataset provides a century-long benchmark for evaluating fire-enabled ecosystem models and supports improved characterization of biomass carbon partitioning between immediate CO_2_ release and longer-lived PyC formation in China.

## Results

### Temporal dynamics of PyC in China

**Figure 1:**
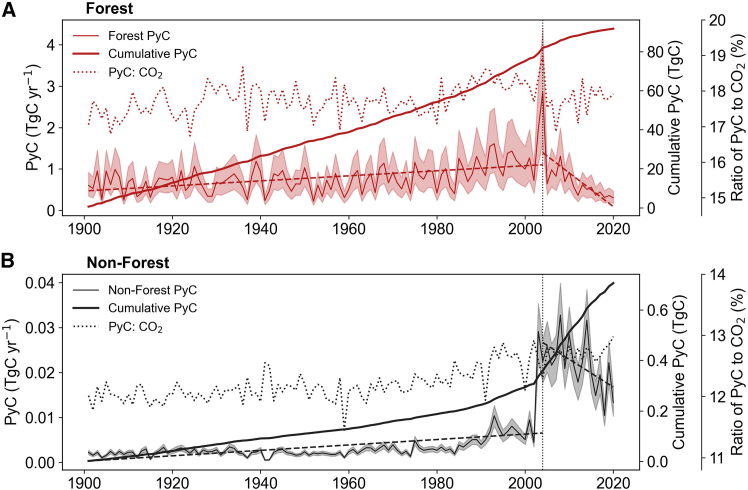
Annual total PyC production estimates for forest and non-forest fires in China during 1901–2020 (A and B) Annual PyC production from forest fires and non-forest fires. The shaded area represents the uncertainty range resulting from the 95% confidence interval (CI) through the Monte Carlo simulations. Dashed lines indicate linear regression trends.

Throughout the study period, annual PyC production for both forest and non-forest fires exhibited substantial interannual variability (Figure 1). Over 1901–2020, the annual PyC production was $0.73_{-0.21}^{+0.22}$ TgC yr^−1^ (median value and interquartile range across years) for forest fires and $0.0028_{-0.0006}^{+0.0023}$ TgC yr^−1^ for non-forest fires (Figure 1). The interannual dynamics of PyC production generally varied with burned area (Figures 1 and S2), highlighting the dominant role of burned area in driving PyC formation. Although the burned areas of forest and non-forest fires were comparable, mean annual PyC production from forest fires was approximately 16.6–857.1 times higher than from non-forest fires (Figures 1 and S2C). This large disparity is mainly attributable to the much higher fuel loads in forest ecosystems ($3346.6_{-628.7}^{+683.2}$ Tg DM yr^−1^), which include substantial woody carbon stocks, compared with the lower fuel loads in non-forest ($23.4_{-8.36}^{+17.2}$ Tg DM yr^−1^) (Figure S3). PyC production for both forest and non-forest fires showed a significantly increasing trend from 1901 to 2004 (forest: slope = 0.0061 TgC yr^−2^; non-forest: slope = 6.0 × 10^−5^ TgC yr^−2^; *p* value <0.05), followed by a significantly decreasing trend from 2004 to 2020 (forest: slope = −0.081 TgC yr^−2^; non-forest: slope = −6.2 × 10^−4^ TgC yr^−2^; *p* value <0.05) (Figure 1). The temporal changes in PyC production are mainly driven by annual changes in burned area (Figure S2C).

The ratio of PyC to fire CO_2_ emissions (PyC:CO_2_) reflects the relative magnitude of PyC production compared with fire-related CO_2_ losses. From 1901 to 2020, this ratio exhibited pronounced interannual variability, largely driven by changes in burned area, fuel load, and combustion completeness. On average, PyC production from forest and non-forest fires is equivalent to 17.8% ± 0.4% (mean annual value ±standard deviation over 1901–2020) and 12.3% ± 0.3% of their respective fire CO_2_ emissions. Although these fractions are relatively small, the long-term persistence of PyC transfers a portion of fire-related biomass carbon into a longer-lived carbon pool, thereby delaying its return to the atmosphere. By considering the decomposition rates of labile and stable PyC using a two-pool exponential decay model (see STAR Methods and Figure S4), the cumulative PyC production increased steadily over 1901–2020, reaching 78.6 TgC for forest fire and 0.62 TgC for non-forest fire by 2020 (Figure 1). This accumulation reflects the prolonged residence time of PyC within terrestrial ecosystems, highlighting its influence on the temporal dynamics of carbon cycling rather than implying a compensatory offset of fire emissions (Figure S5).

### Spatial patterns of PyC in China

**Figure 2:**
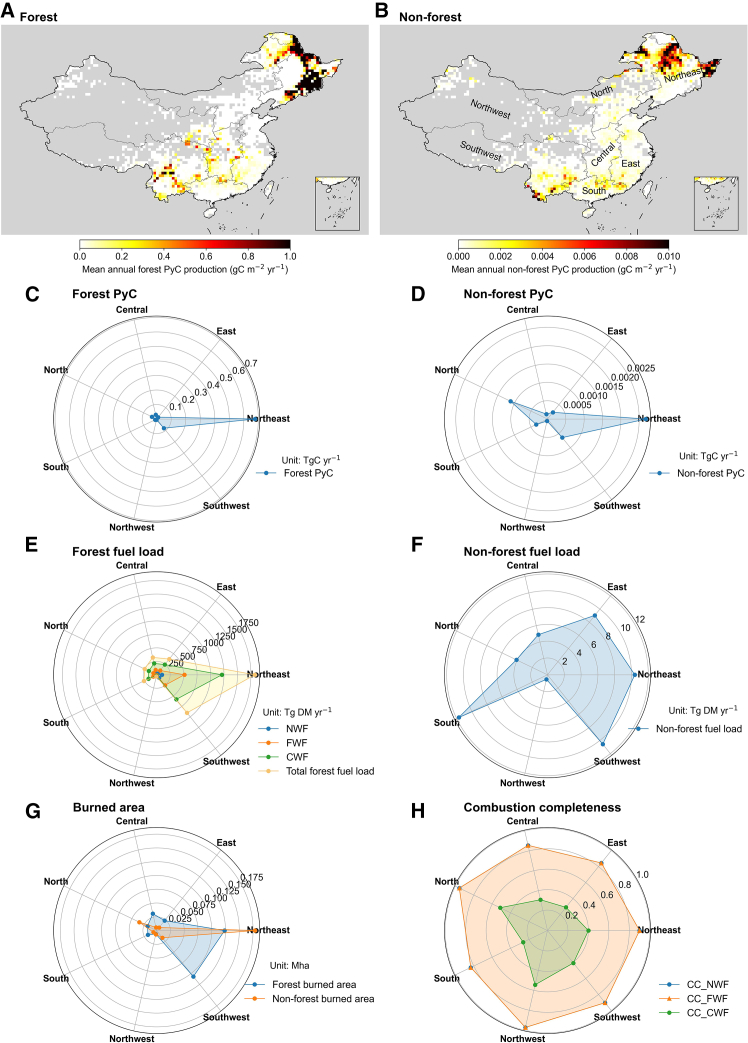
Spatial distribution and regional characteristics of PyC production in China during 1901–2020 (A and B) Spatial distribution of mean annual PyC production from forest fires (A) and non-forest fires (B). (C and D) Mean annual regional total PyC production from forest (C) and non-forest fires (D) across seven regions of China over 1901–2020. (E and F) Mean annual regional total fuel loads for forest (E) and non-forest (F). (G) Mean annual regional total burned area for forest and non-forest fires. (H) Mean annual regional mean combustion completeness across seven regions. NWF, FWF, and CWF refer to non-wood fuel, fine wood fuel, and coarse wood fuel, respectively.

The spatial distribution of mean annual PyC production for forest and non-forest fires over 1901–2020 exhibited distinct regional patterns (Figures 2A and 2B). Forest PyC production was overwhelmingly concentrated in the Northeast (0.68 TgC yr^−1^)—particularly in the *Lesser Khingan* and *Changbai Mountains* (Figure S6)—and, to a lesser extent, the Southwest (0.08 TgC yr^−1^), accounting for 79.0% and 9.4% of the national total, respectively. In contrast, non-forest PyC production was substantially lower overall, with the Northeast (54.5%), North (20.5%), and Southwest (11.5%) contributing the largest shares (Figures 2A and 2B). These spatial patterns were closely linked to fuel load, burned area, and fuel type. For forest fires, the highest biomass fuel loads and burned areas in the Northeast led to substantial PyC accumulation (Figure 2). Among fuel types, coarse wood fuel (CWF) contributed the most to PyC production (Figure 2E). Moreover, the relatively larger CWF-specific PyC production factor (Table S2), together with lower combustion completeness (CC, reflecting more incomplete combustion), further promoted PyC formation in the Northeast. In contrast, the relatively lower PyC production in the Southwest may be attributed to a lower proportion of fuels on burned area, which tend to burn more completely owing to higher CC associated with favorable hydrothermal conditions in temperate or subtropical forests (Figure 2). For non-forest fires, although fuel loads in the Northeast and North were not the highest, their larger burned areas led to substantial PyC production. Conversely, the East, Southwest, and South exhibited higher fuel loads but smaller burned areas, resulting in lower PyC production (Figure 2).

**Figure 3:**
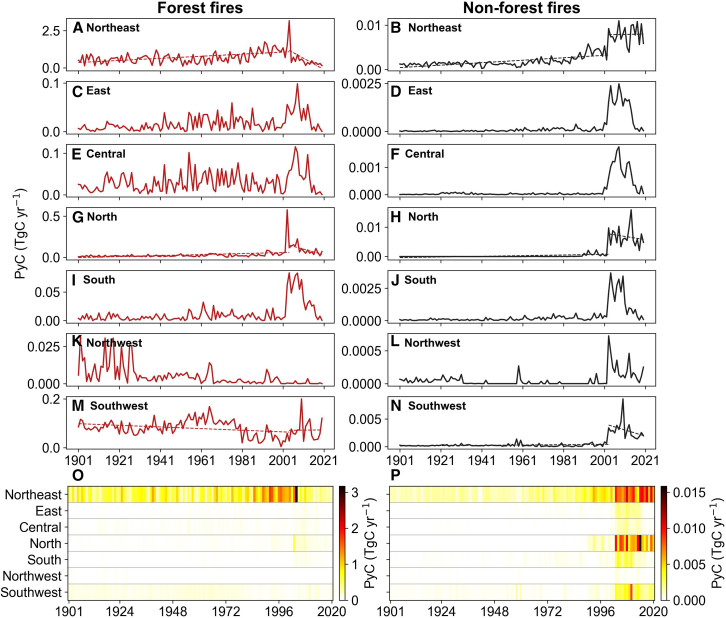
Annual PyC production in seven regions of China during 1901–2020 Images are arranged by region from top to bottom. The left column shows PyC production from forest fires (A, C, E, G, I, K, M, and O), and the right column shows PyC production from non-forest fires (B, D, F, H, J, L, N, and P). Dashed lines indicate linear regression trends.

Across all regions, PyC production from both forest and non-forest fires exhibited pronounced interannual variability, largely driven by variations in burned area and fuel load (Figure 3; Figures S7 and S8). Over the whole studied period of 1901–2020, the Northeast consistently dominated forest PyC production and showed a significant increasing trend from 1901 to 2004 (slope = 0.007 TgC yr^−2^; *p* value <0.05), followed by a significant declining trend from 2004 to 2020 (slope = −0.076 TgC yr^−2^; *p* value <0.05). Peak forest PyC production in the Northeast reached 3.2 TgC yr^−1^ in 2004, far exceeding that of all other regions (Figure 3), which generally remained below 0.6 TgC yr^−1^, thereby contributing the largest to the national total increase (Figure 2). Specifically, forest PyC production in the North was relatively small but increased sharply to a peak of 0.58 TgC yr^−1^ in 2003, associated with major forest fire events.

For non-forest fires, the Northeast (slope = 2.8 × 10^−5^ TgC yr^−2^; *p* value <0.05) and North (slope = 1.1 × 10^−5^ TgC yr^−2^; *p* value <0.05) contributed most to the increasing trend in the national total PyC production during 1901–2003, followed by the Southwest (Figure 3). However, the declining trends in PyC production from 2003 to 2020 in these regions were not statistically significant (*p* value >0.05), with peak values of 0.011 TgC yr^−1^ in 2008 in the Northeast, 0.016 TgC yr^−1^ in 2014 in the North, and 0.009 TgC yr^−1^ in 2010 in the Southwest, respectively (Figure 3). By contrast, the significant decrease in national total PyC production during 2004–2020 is primarily attributed to declines across the remaining regions. Overall, these results highlight strong regional heterogeneity in PyC production, with the Northeast acting as a persistent hotspot for both forest and non-forest fires and the Northeast and North dominating non-forest PyC formation since the 21st century (Figures 3 and S9).

### Uncertainty analysis

**Figure 4:**
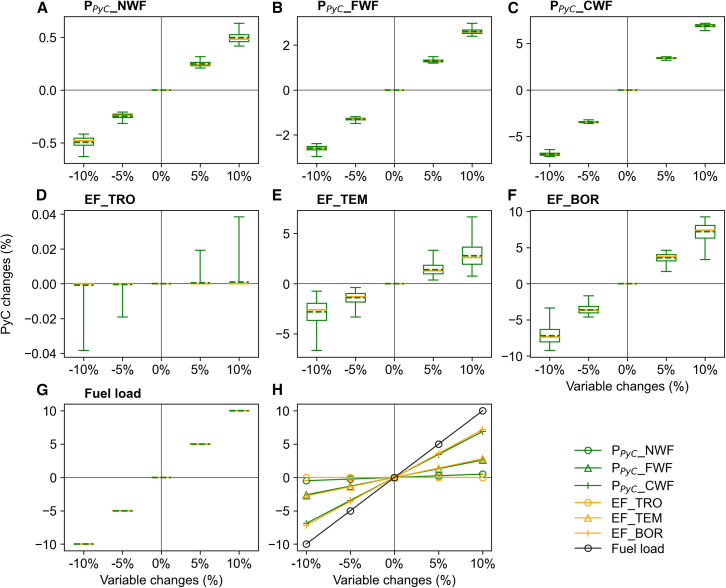
Sensitivity of national total PyC production to key variables under different perturbation levels over 1901–2020 (A–C) Responses to perturbations in PyC production factors for non-wood fuels (P_PyC__NWF), fine wood fuels (P_PyC__FWF), and coarse wood fuels (P_PyC__CWF). (D–F) Responses to perturbations in CO_2_ emission factors for tropical PFTs (EF_TRO), temperate PFTs (EF_TEM), and boreal PFTs (EF_BOR). (G) Response to perturbations in fuel load. Yellow lines and dashed green lines in the boxplots indicate the median and mean of the annual national total PyC changes under perturbations during 1901–2020, respectively. (H)Mean annual changes in national total PyC production under four perturbation levels.

By considering the variability in key variables across different plant functional types (PFTs) and fuel types, including fuel load, dry matter carbon content (DMCC), fire CO_2_ EFs, and PyC production factors (Table S3), we estimated the mean annual PyC production to be 0.8 TgC yr^−1^ for forest fires, ranging from 0.4 TgC yr^−1^ to 1.2 TgC yr^−1^, and 0.0059 TgC yr^−1^ for non-forest fires, ranging from 0.0046 TgC yr^−1^ to 0.0071 TgC yr^−1^ (Figure 1). Among all parameters, fuel load exerted the greatest influence, highlighting the critical role of fuel availability in controlling PyC formation (Figure 4). PyC production factor for coarse wood fuels (P_PyC__CWF), combustion completeness for coarse wood fuels (CC_CWF, which shared sensitivity with P_PyC__CWF, see STAR Methods), and the CO_2_ emission factor for boreal PFTs (EF_BOR) exhibited higher sensitivity than other PFTs and fuel types, indicating that boreal forest types and coarse woody components are the dominant contributors to PyC production. In contrast, the sensitivity to the PyC production factor for non-wood fuels (P_PyC__NWF) and the CO_2_ emission factor for tropical PFTs (EF_TRO) was relatively low. Notably, the sensitivities of CO_2_ EFs in temperate and boreal forests exhibited greater interannual variability than other parameters.

## Discussion

### Comparison with other studies

Current studies provide only limited estimates of PyC production in China. Global PyC production has been estimated at 49.5–153.4 TgC yr^−1^ for 2000–2010,^15^ 256 TgC yr^−1^ for 1997–2016,^9^ and 337 TgC yr^−1^ for 1901–2010.^8^ These differences are largely driven by the modeling approaches employed. For example, the global PyC production estimated from the GFED4s fire emission inventory (153.4 ± 18.7 TgC yr^−1^) is approximately three times higher than that derived from the terrestrial ecosystem model version 6 (TEM6; 49.5 ± 4.9 TgC yr^−1^).^15^ Our estimate of PyC production in China (0.8 TgC yr^−1^, ranging from 0.4 to 1.2 TgC yr^−1^) represented a small fraction of global PyC production, primarily because China accounts for a relatively small share of global burned area. However, the spatial patterns of PyC production in China derived from the global fire-based PyC estimate show substantial discrepancies when compared with burned area inventories^8^ (Figure S10). The large variations among different global estimation approaches further highlight the challenges in accurately quantifying PyC production at regional scales.^15^

Given the scarcity of reliable PyC estimates in China, direct validation of our PyC estimates remains difficult. Therefore, we compared our estimated fire CO_2_ emissions with previous studies as an additional consistency check, considering the close linkage between PyC formation and fire CO_2_ emissions. Estimates of PyC and associated fire CO_2_ emissions can vary substantially across studies because they rely on different methodological choices and key input datasets, particularly burned area and biomass. Our estimated annual average forest fire CO_2_ emissions over 2003–2017 is 5.9 TgC yr^−1^ (Figure S11), which is comparable to the estimate of 6.3 TgC yr^−1^ reported by Gong et al.,^20^ but lower than the 11.1 TgC yr^−1^ estimated by Yin et al.^26^ for the same period, and the 9.9 TgC yr^−1^ reported by GFED4s for 2003–2016.^20^ These discrepancies likely arise from differences in biomass inputs and burned-area datasets, which present major sources of uncertainty. For non-forest fires, our estimate of mean annual CO_2_ emissions during 2003–2017 (0.19 TgC yr^−1^, Figure S11) is slightly lower than the 0.25 TgC yr^−1^ reported by Gong et al.,^20^ and substantially lower than the estimates of 3.85 TgC yr^−1^ from Yin et al.^26^ and 5.37 TgC yr^−1^ from GFED4s at the same period. These differences likely reflect variations in land-cover maps and non-forest definitions. While some studies include agricultural burning on croplands, we restricted non-forest fires to natural grass PFTs and excluded all managed land covers.

Regionally, the Northeast and Southwest exhibited higher PyC production (Figure 2), consistent with the patterns observed in ground-based surveys of forest fire number and burned area across China.^27^ In these regions, climate and vegetation variables are the primary drivers of forest fires,^27^^,^^28^ particularly in the Northeast, where frequent, large-scale forest fires and abundant coarse wood fuel availability promote substantial PyC formation. In addition, topographic variables (e.g., elevation, slope, and aspect) and socioeconomic variables (e.g., population density and gross domestic product) play a prominent role in the Southwest, reflecting higher fire occurrence probabilities associated with vegetation distribution and intensive human activities.^27^^,^^29^ Over the long term, the more frequent and higher PyC peaks in the past two decades may be partly associated with intensified drought under climate warming, which enhances fuel flammability and promotes large-scale forest fires. The declining trend in PyC production from forest fires since 2004 (Figure 1) is consistent with statistical decreases in both the annual number of fires and burned area,^18^ with the largest burned forest area occurring in the Northeast.^30^ The decreasing trend may be attributed to the implementation of forest fire prevention and strengthened control polices in China.^31^ Overall, the consistency of fire CO_2_ estimates with previous inventories, as well as the use of observed PyC:CO_2_ ratios, support the reliability of our PyC production estimates for China.

### Uncertainties

The estimates of fire CO_2_ emissions and subsequent PyC production strongly depend on the quality of the input burned area and biomass datasets. Although biomass density simulated by the ORCHIDEE model was calibrated against field observations, substantial uncertainties in fuel load estimates may still arise due to differences in biogeochemical processes and parameterizations across dynamic global vegetation models. For example, multi-model simulations from the TRENDY project showed a wide range of forest biomass carbon density estimates for China, with a variability of approximately 18%, reflecting differences in the representation of land biophysical processes and biogeochemical cycles^32^ (Figure S12). Although biomass estimates derived from field observations, forest inventories, and remote sensing products may offer higher accuracy at local to regional scales, these data are often limited in spatial coverage or lack long-term consistency, making them incapable of supporting century-scale analyses. To ensure spatiotemporal consistency across the entire study period, we therefore relied on model-simulated biomass data, which provide a unified long-term representation of biomass dynamics despite inherent uncertainties. In addition, our assumption on the fixed allocation fraction of biomass to fuel classes may not explicitly capture regional differences in vegetation structure, successional stage, or climate-driven variability in fuel composition. Such variability may influence combustion completeness and PyC formation efficiency, potentially introducing additional uncertainty into fire emissions and PyC estimates. Future studies incorporating spatially dynamic fuel structure information would further reduce uncertainty.

Burned area is another key determinant of PyC production. In our approach, the total burned area fraction within each grid cell was allocated to vegetation types according to their fractional coverage. This may introduce uncertainties because actual burned fractions often differ among PFTs due to differences in vegetation characteristics, fire behavior, and environmental conditions. For instance, variations in species composition, stand structure, and surface fuel conditions can lead to substantial differences in fire behavior and burned fractions across forest subtypes and locations.^33^^,^^34^

We used PFT-specific parameters (e.g., DMCC and EF) and fuel-type-specific parameters (e.g., combustion completeness, PyC production factor) to better represent the variability relevant to PyC estimates. However, uncertainties remain because several key parameters (e.g., PyC production factor, combustion completeness) may vary across both biomass components and ecosystem levels, but these variations are not fully captured in our study. Although our sensitivity analyses indicated that PyC production responds differently to parameter changes across PFTs and fuel types (Figure 4), many of the parameters used are available only at the PFT or fuel-type level. For example, PyC production factors vary not only among fuel classes (i.e., NWF, FWF, and CWF in this study) but also across PFTs and fire types defined by ignition sources and land-use context (e.g., wildfires and deforestation fires).^15^^,^^35^ EF and DMCC are PFT specified, but they are assigned according to broad bioclimatic classes (tropical, temperate, boreal, and savanna; Table S1) in this study. This aggregation may not fully capture intra-regional or PFT-specific variability of PyC production, particularly in ecologically heterogeneous landscapes. In addition, PyC formation is influenced by weather conditions (e.g., drought) and fire characteristics, including fire temperature, duration, and spread speed.^15^^,^^36^ Combustion completeness also differs across PFTs and climate zones,^22^^,^^37^ which is also influenced by local topography, weather conditions, and vegetation composition.^24^ Our representation of CC as linearly scaled with soil moisture simplifies potentially non-linear combustion responses under extreme moisture conditions, and does not explicitly account for regional differences in fire regimes. Nevertheless, this parameterization follows the widely adopted GFED framework and enables a spatially and temporally explicit representation of CC over long time periods in the absence of detailed observational constraints. Future work should incorporate more regionally differentiated parameterizations to improve model accuracy.

Moreover, due to limited data availability and inconsistencies in national-scale inventories, fires occurring in peatlands, managed ecosystems (e.g., croplands and pastures), and other biomass types (e.g., mosses) were not included in this analysis, potentially leading to an underestimation of the total PyC production in China. According to GFED4s, fire CO_2_ emissions from peatlands and agricultural lands in China over 2005–2015 account for approximately 0.03 TgC yr^−1^ and 9.55 TgC yr^−1^, respectively, together contributing 39.8% of total fire CO_2_ emissions across all land-cover types (Figure S13). While CO_2_ emissions cannot be directly translated into PyC production because of differences in combustion conditions and PyC formation efficiency, these proportions suggest that excluding these land-use types could contribute to uncertainty in national PyC estimates. Future studies should incorporate these land-use types explicitly and validate estimates using ground observations, higher-resolution regional inventories, or scenario analysis to better constrain their contributions. Beyond improved data coverage, future improvements should refine the biogeochemical representation of fire behavior across different ecosystems by incorporating key influencing factors, such as burn severity, climate conditions, and vegetation structure, into the parameterization of fire CO_2_ emissions factors. This would help better constrain region-specific parameters and reduce uncertainties in PyC estimates.

### Implications

Due to its high chemical stability, PyC production may constitute an important component of the global carbon cycle.^9^^,^^10^^,^^13^ In addition to altering the fate of fire-derived carbon, PyC retained in soils can influence soil carbon dynamics and affect a range of biogeochemical processes.^14^^,^^38^^,^^39^ For example, prescribed burning has been shown that PyC can enhance soil microbial biomass, soil organic carbon (SOC), and nutrient cycling in boreal ecosystems.^40^ PyC has been estimated to account for up to 40% of surface SOC in African savannas based on extensive soil PyC measurements.^41^ However, the long-term degradation characteristics of PyC remain uncertain. Although PyC is generally viewed as a long-term carbon pool, studies have shown that some fraction of PyC can be mineralized over a much shorter timescale of weeks.^10^ Additional losses may also occur through reburning, and this reburning flux is equivalent to approximately 9%–56% of global PyC decomposition fluxes.^42^

PyC decomposition is also influenced by temperature, particularly under global warming.^43^ However, this effect was not explicitly considered in our estimates of the long-term carbon sequestration potential of PyC due to the large uncertainties involved. Due to limited data and broadly similar formation processes, the decomposition parameters used in the two-pool model are primarily derived from biochar incubation studies. However, the stability of wildfire-derived PyC may differ from that of engineered biochar due to variations in feedstock type, combustion conditions, post-fire environmental processes, and subsequent disturbance such as erosion or reburning.^42^^,^^44^ Therefore, our estimates of PyC persistence, which rely on the transferability of these parameters, likely represent a first-order approximation under current data constraints. Improved field-based measurements of wildfire-derived PyC turnover rates across different ecosystems and climate regimes would help constrain decomposition parameters and reduce associated uncertainties. Moreover, the darkened postfire surface substantially reduces soil albedo and induces local surface warming through biophysical feedbacks.^45^^,^^46^ Therefore, both the long-term biogeochemical behavior of PyC and its biophysical climate impacts should be explicitly considered when incorporating PyC into regional and global carbon budgets. From an environmental perspective, various pollutants co-produced during PyC formation may also pose risks to ecosystem functioning and human health.^38^^,^^47^ Their formation mechanisms and potential toxicological effects on organisms require further investigation.

Under future climate change, increased fire activity is expected to alter both the magnitude and spatial distribution of PyC formation.^48^ Changes in fire regimes may influence biomass consumption and combustion conditions, thereby affecting PyC production pathways. For example, climate warming and altered precipitation patterns can lead to longer and more fire-conducive seasons, which enhance fuel flammability and fire intensity, potentially increasing PyC formation in some ecosystems.^49^ These shifts in fire weather and behavior may alter the balance between immediate CO_2_ release and longer-lived carbon storage in PyC pools. These projections underscore the need to incorporate PyC into fire-related carbon accounting and to strengthen fire prevention and management, particularly in PyC hotspot regions such as Northeast and Southwest China (Figure 3). Understanding how PyC production may respond to climate-driven shifts in fire activity remains an important direction for future research. In addition, continued advancements in process-based models are crucial for more accurately representing fire occurrence, spread dynamics, and associated biogeochemical impacts on PyC pools. Such advancements will enhance our ability to predict future fire regimes and associated risks.

Overall, this study provides a century-long, spatially explicit quantification of the magnitude and spatiotemporal patterns of PyC production from forest and non-forest fires in China from 1901 to 2020, providing a refined estimate based on reconstructed long-term burned area data. Our results show that Northeast and Southwest China are the major contributors to PyC production from forest fires, while the North additionally contributes to non-forest PyC production, and that the national total PyC has declined since ∼2004. Given its high stability, PyC presents a potentially long-term carbon pool, which should be incorporated into future Earth system models to constrain terrestrial carbon budgets and fire-related carbon partitioning between immediate CO_2_ emissions and longer-term PyC storage. Moreover, the drivers of PyC formation and the mechanisms through which PyC influences soil carbon cycling require further investigation under future climate change.

### Limitations of the study

This study has several limitations, primarily related to data availability and model assumptions. First, uncertainties in simulated long-term biomass datasets propagate into PyC estimates despite model calibration. Second, simplified assumptions on burned area allocation, fuel partitioning, and combustion processes may not fully capture spatial variability in vegetation structure and fire behavior. Third, key parameters such as combustion completeness and PyC production factors are applied at aggregated fuel-type levels, potentially overlooking ecosystem-specific variability. Finally, fires in peatlands and managed ecosystems were not included, which may lead to an underestimation of total PyC production. Future work incorporating spatially explicit datasets and improved process-based parameterizations would help enhance the accuracy of PyC estimates.

## Resource availability

### Lead contact

Requests for further information and resources should be directed to and will be fulfilled by the lead contact, Mengjie Han (hanmengjie@tsinghua.edu.cn).

### Materials availability

This study did not generate new unique reagents.

### Data and code availability

• Data: The data supporting the findings of this study are available within the manuscript and the supplemental information.
• Code: The codes used for data processing and analysis are available at Zenodo: https://doi.org/10.5281/zenodo.18908534.
• Additional information: Any additional information required to reanalyze the data reported in this paper is available from the lead contact upon request.

## Acknowledgments

This study was funded by the 10.13039/501100001809National Natural Science Foundation of China (grant nos.: 42407643, 42307496, and 42571109) and the Yunnan Provincial Science and Technology Project at Southwest United Graduate School (grant no.: 202302AO370001). This study was supported by the Fundamental Research Funds for the Central Universities (grant no.: 2652022046), the Tsinghua University Dushi Program, and the Center of High-Performance Computing, Tsinghua University.

## Author contributions

All authors contributed to the study conception and design. Material preparation, data collection, and analysis were performed by C.Y., M.H., and W.L. The first draft of the manuscript was written by C.Y. and M.H., and all authors commented on previous versions of the manuscript. All authors read and approved the final manuscript.

## Declaration of interests

The authors declare no conflict of interest.

## STAR★Methods

### Key resources table

**Table:** 

REAGENT or RESOURCE	SOURCE	IDENTIFIER
**Deposited data**		

Code for the process and analysis of PyC production	This paper	https://doi.org/10.5281/zenodo.18908534
Burned area data	Guo et al.^25^	https://doi.org/10.5194/essd-17-3599-2025
Biomass data	Leng et al.^17^	https://doi.org/10.1016/j.oneear.2024.04.011

**Software and algorithms**		

Python (version 3.7.0)	Python Software Foundation	https://www.python.org
Numpy	NumPy Developers	https://numpy.org
SciPy	SciPy Developers	https://scipy.org
Pandas	Pandas Developers	https://pandas.pydata.org

### Method details

#### Calculation of PyC production

According to the GFED4s methodology,^16^ we estimated fire-derived CO_2_ emissions using a bottom-up approach based on burned area and fuel consumption per unit of burned area. Pyrogenic carbon (PyC) production was then quantified using a PyC production factor, defined as the amount of PyC generated per unit of CO_2_-C emitted.^9^ Accordingly, the PyC production was calculated using Equation 1:

(Equation 1) $$PyC=\sum_{i=1}^{\text{PFT}}\sum_{j=1}^{\text{Fuel}}\;\left(\frac{FL_{i,j}}{DMCC_{i}}\times BA_{i}\times CC_{j}\times EF_{i}\times \frac{12}{44}\times P_{PyCj}\right)$$

where *i* and *j* are the indices of plant functional type (PFT) and fuel type, respectively (Table S1). FL is the biomass fuel load (gC m^−2^); BA is the burned area (m^2^); CC is the combustion completeness; EF is the CO_2_ emission factor (g CO_2_ kg^−1^ dry matter (DM) burned); DMCC is the carbon content of DM (%); *P*_*PyC*_ is the PyC production factor, defined as an empirical ratio that quantifies the amount of PyC per unit of fire-related CO_2_ emissions. It represents the partitioning of burned biomass between complete oxidation (CO_2_ emissions) and incomplete combustion that leads to PyC formation, rather than a direct conversion of CO_2_ into PyC. 12/44 is the conversion factor from CO_2_ to C. Among these variables, EF and DMCC are PFT-specific, whereas CC and *P*_*PyC*_ are fuel-type-specific.

All calculations were conducted at the grid-cell scale and national total PyC production was derived by summing across all burned grid cells. Because individual grid cells may contain multiple PFTs, sub-grid processing was implemented to explicitly account for differences in vegetation type and fuel composition (Figure S1). In each grid cell, biomass and burned area are partitioned among coexisting PFTs according to their fractional coverage, and fuel types are defined within each PFT based on biomass components (Figure S1). Thus, fuel types are nested within PFTs, and total PyC production for each grid cell is obtained by summing over all PFT and fuel type combinations without overlap. Note that each combination was already associated with its absolute area within the grid cell, so the summation was performed directly without weighting by fractional coverage.

Notably, standard fire emission inventories (e.g., GFED4s) typically quantify CO_2_ emissions from the oxidized fraction of burned biomass. In our framework, PyC represents the non-oxidized carbon fraction formed through incomplete combustion and is therefore not included in reported fire CO_2_ emissions. Our approach explicitly characterizes this partitioning at the time of combustion by applying the PyC productor factor, thereby distinguishing between immediate CO_2_ release and carbon retained as PyC, rather than implying any subsequent removal or compensation of emitted CO_2_ (Figure S5).

#### Burned area

We obtained the monthly century-scale (1901–2020) burned area data for China at 0.5 ° × 0.5 ° spatial resolution from Guo et al.^25^ To ensure consistency with the annual temporal resolution of our PyC production estimates, the monthly burned area data were aggregated to annual totals. Although this aggregation does not explicitly resolve fire seasonality, our analysis focuses on long-term interannual variability in PyC production. As PyC formation is assumed to scale proportionally with total burned area, the use of annual aggregates is unlikely to substantially affect the magnitude of the estimated annual PyC fluxes. This dataset was reconstructed using a machine learning model trained on satellite-derived burned area observations and incorporating climatic, vegetation, and human activity variables. The model performed well in reproducing long-term spatial patterns and interannual variability (R^2^ = 0.69–0.98). Based on this dataset, we analyzed two types of forest and non-forest fires, which represent natural vegetation types and exclude all managed land covers. For non-forest, only natural C3 and C4 grass PFTs were retained (Table S1), while managed PFTs, including C3 pasture, C4 pasture, C3 cropland, and C4 cropland, were excluded from our analysis. The spatiotemporal distribution of burned area for forest and non-forest ecosystems is shown in Figure S2.

#### Fuel loading

We obtained annual vegetation biomass data for China over 1901–2020 from Leng et al.^17^ as the basis for estimating potential fuel loads. These data were simulated using the process-based dynamic vegetation model ORCHIDEE-MICT,^50^ which explicitly incorporates key processes such as forest age cohorts and wood harvest and has been calibrated against field-observed aboveground biomass in China.^17^ As a result, the model performs well in reproducing total aboveground biomass and forest carbon fluxes in China,^17^ providing confidence in the reliability of these biomass estimates for use in this study.

The biomass dataset is provided by plant functional type (PFT) and biomass components at a spatial resolution of 0.5 ° × 0.5 °. To match the burned-area classification of Guo et al.,^25^ we included only natural PFTs, excluding cropland and pasture from the analysis. After this exclusion, eight forest PFTs and two grass PFTs remained (Table S1). For each PFT, we assumed that only aboveground biomass can burn, and thus we incorporated four aboveground biomass components—leaf, aboveground sapwood, aboveground heartwood, and fruit—into the fuel calculation (Table S2).

The biomass components were further classified into four fuel types of 1-h, 10-h, 100-h, and 1000-h fuels, which are based on stem diameter and the corresponding moisture loss timescale under idealized atmospheric conditions.^8^ All leaves and fruits were partitioned into 1-h fuels, whereas 4.5%, 7.5%, 21% and 67% of aboveground sapwood or aboveground heartwood were assigned to the 1-h, 10-h, 100-h, and 1000-h fuels classes, respectively^8^ (Table S2). To align with the fuel classification scheme of the PyC production factors, these four fuel types were reallocated into three categories: non-wood fuel (NWF), fine woody fuel (FWF), and coarse woody fuel (CWF) (Table S2). Specifically, 1-h fuels (mainly leaves) were grouped as NWF, 10-h (small branches) and 100-h fuels (large branches) were grouped as FWF, and 1000-h fuels (tree trunks) were classified as CWF.^8^ The spatiotemporal distribution of total fuel load across these four classes within burned areas is shown in Figure S3. For each grid cell, the fuel burned by each PFT was determined by its corresponding fuel load and burned area, where the total burned area of forest or non-forest within each grid cell was partitioned among PFTs in proportion to their fractional coverage.

In this study, we assumed that all aboveground biomass is potentially combustible and that the allocation fractions of biomass components to different fuel classes (1-h to 1000-h) are fixed in space and time. This simplification is commonly adopted in large-scale fire emission modeling due to the lack of spatially explicit and temporally resolved fuel-structure datasets.^8^ In addition, due to the lack of sub-grid information on fire occurrence, we assumed that the probability of burning is proportional to the fractional coverage of each vegetation type within a grid cell, without explicitly accounting for potential differences in fire susceptibility among vegetation types.

#### Combustion completeness and emission factor

Combustion completeness (CC) is a key parameter in fire emission calculations, representing the fraction of available fuel load that is actually consumed during a fire. The parameter exhibits substantial spatial variability because it is affected by combustion processes, fire severity, fuel type, and climatic conditions such as humidity.^21^^,^^51^ In this study, the minimum and maximum CC were set for each fuel type following van der Werf et al.^52^ (Table S2). CC was then linearly scaled using soil moisture, such that the 5^th^ and 95^th^ percentiles of soil moisture correspond to the minimum and maximum CC values, respectively.^16^ Annual soil moisture data for 1901–2020 were obtained from ORCHIDEE-MICT simulations and used to produce the spatially explicit CC parameter across China.

For emission factors (EF) and dry matter carbon content (DMCC), which were typically assigned based on vegetation types, we adopted the values for tropical, temperate, boreal, and savanna ecosystems in GFED4.^16^ The EF and DMCC values were then mapped to the corresponding PFTs used in this study (Table S1).

#### PyC production factor

We used the PyC production factor database developed by Jones et al.,^9^ which was derived from literature synthesis and defined as the ratio of PyC produced during fire to the CO_2_-C emitted (PyC:CO_2_). Here, the emitted carbon refers to the amount of carbon actually combusted and is calculated as pre-fire carbon minus the sum of postfire remaining carbon and PyC, thereby explicitly excluding PyC from the combusted carbon pool. These PyC production factors are fuel-type specific and categorized into three classes of NWF, FWF, and CWF. Following Bowring et al.,^8^ the four biomass fuel classes (1-h, 10-h, 100-h, and 1000-h fuels) were further aggregated into three fuel categories (NWF, FWF, and CWF) based on fuel characteristics (Table S2). It should be noted that fuel-type-specific parameters are implicitly PFT-dependent because fuel type is embedded within each PFT. Although identical fuel-type-specific parameter values were used across PFTs sharing similar fuel categories, differences in biomass allocation and fuel composition among PFTs resulted in distinct PyC production at the grid-cell scale (Figure S1). The mean estimates and bootstrapped 95% confidence interval (CI) of the PyC production factors reported by Jones et al.^9^ were used to estimate PyC production in China (Table S2).

#### Analyses of PyC production in China

Using Equation 1, we calculated annual PyC production for forest and non-forest fires at the grid-cell level and aggregated grid-level estimates to obtain the national total PyC production for China. We first analyzed the interannual dynamics of forest and non-forest PyC production from 1901 to 2020 and assessed its magnitude relative to fire-induced CO_2_ emissions in China.

In order to assess the long-term carbon sequestration potential of PyC, we quantified the annual cumulative PyC production for forest and non-forest fires by explicitly considering PyC decomposition. We applied a two-pool exponential decay model that partitions PyC into labile and stable fractions, each characterized by distinct decomposition rates (Equation 2). Parameter values were adopted from Landry & Matthews.,^42^ who derived these parameters based mostly on field observations and incubation experiments of biochar, while considering differences in decomposition rate and experimental conditions (e.g., field, laboratory) between biochar and naturally produced PyC. Notably, biochar generally exhibits lower turnover rates than naturally produced PyC, and decomposition rate measured in laboratory experiments tends to be faster than that under field conditions.^42^ The decomposition curve of PyC is shown in Figure S4.

(Equation 2) $${PyC}_{remain}=a\times e^{\left(-k_{l}\times t\right)}+b\times e^{\left(-k_{s}\times t\right)}$$

where *PyC*_*remain*_ presents the percentage of the initial PyC remaining (%), *t* is time (year), *a* and *b* are the fractions of labile and stable PyC, respectively, with values of 9.1% and 90.9%. *k*_*l*_ and *k*_*s*_ denote the decomposition rates of the labile and stable PyC pools, with values of 0.5 years^−1^ and 1.1 × 10^−3^ yr^−1^, respectively.

We then investigated the spatiotemporal distribution of PyC across seven subregions (i.e., Northeast, East, Central, North, South, Northwest, Southwest; Figure S6). Finally, we quantified parameter-driven uncertainty and identified key controls on PyC production through the Monte Carlo and sensitivity analyses.

#### Uncertainty and sensitivity analysis

Considering the uncertainties in simulated biomass and the diversity of PFTs and fuel types, which lead to variations in dry matter carbon content, EF, and PyC production factors, we used Monte Carlo simulations to estimate the uncertainty range of PyC production for forest and non-forest fires. Random values were generated from assumed normal distributions (Table S3), and after 1000 iterations, the 95% confidence intervals (95% CI) were calculated as the uncertainty range. While some parameters (e.g., PyC production factor) are physically bounded and may exhibit skewed distributions, the normal approximation provides a practical and widely adopted approach for large-scale uncertainty estimation in the absence of detailed distributional information for most parameters.^53^^,^^54^ In addition, given that the coefficients of variation for these parameters are relatively small (Table S3), the influence of the distribution shape on the resulting 95% confidence intervals is expected to be limited.

Importantly, parameters were assumed to be statistically independent due to the lack of empirical constraints on their covariance structures at the national scale. In reality, certain variables (e.g., fuel load and CC) may be partially correlated through shared climatic and ecological controls. Similarly, although CC and P_PyC_ both relate to incomplete combustion processes, they represent distinct components in our framework. CC quantifies the fraction of available biomass that is consumed during burning, whereas P_pyc_ characterizes the proportion of combusted carbon that forms PyC rather than being fully oxidized to CO_2_. While these parameters may co-vary under specific combustion conditions, robust empirical data are currently insufficient to constrain their covariance at large spatial scales. Therefore, assuming independence provides a practical and conservative approach for estimating overall uncertainty.

In addition, a sensitivity analysis was conducted for forest fires by perturbing one parameter at a time while keeping all others constant. The variables analyzed include (1) fuel load, (2) PyC production factors for non-wood, fine wood and coarse wood fuels (P_PyC__NWF, P_PyC__FWF, P_PyC__CWF), (3) combustion completeness for the corresponding fuel types (CC_NWF, CC_FWF, CC_CWF), (4) carbon content in dry matter for different PFTs (DMCC_TOR, DMCC_TEM, DMCC_BOR), and (5) CO_2_ EF for different PFTs (EF_TOR, EF_TEM, EF_BOR) (Table S3). Perturbations of −10%, −5%, 5% and 10% were applied to each parameter. It should be noted that due to the linear structure of the equation (Equation 1), parameters associated with the same fuel type or PFT produce identical changes in PyC estimates. For example, the sensitivity of PyC production to P_PyC__NWF is equivalent to that for CC_NWF.

### Quantification and statistical analysis

For forest and non-forest fires, the temporal trends in PyC production at national and regional scales were assessed using ordinary least squares linear regression for different periods. The regression analyses were conducted using the *linregress* function from the Python package “SciPy”, which provides estimates of the slope and two-sided *p*-value for testing the magnitude and statistical significance of trends. A trend was considered statistically significant when *p* < 0.05. Spatial differences in PyC production among regions were evaluated using descriptive statistics and proportional contributions to the national total. Uncertainty ranges were quantified using Monte Carlo simulations as described in Uncertainty and sensitivity analysis section. All statistical analyses were performed in Python (version 3.7.0), primarily using the packages NumPy, SciPy, and Pandas.
