# Supplementary material for: Pyrogenic carbon production from fires in China from 1901 to 2020
Source: iScience. 2026 May 22;29(6):116060. doi: 10.1016/j.isci.2026.116060 (PMC13223992; doi:10.1016/j.isci.2026.116060)
Supplement: Document S1. Figures S1–S13 and Tables S1–S3 [file mmc1.pdf]

iScience, Volume *29*

Supplemental information

Pyrogenic carbon production from fires in China from 1901 to 2020

Chenyi Yuan, Mengjie Han, Minxuan Sun, Bo Pan, Qing Zhao, and Wei Li

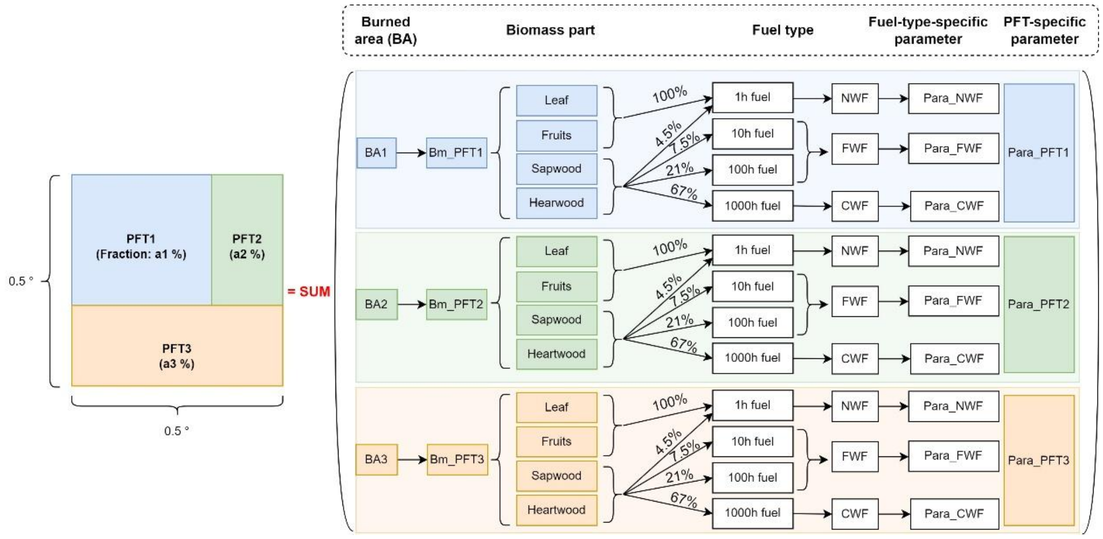

Figure S1. Schematic flowchart illustrating the calculation of PyC production within a single grid cell, Related to STAR Methods.

PFT: plant functional type; BA: burned area; Bm: biomass; NWF: non-wood fuel; FWF: fine woody fuel; CWF: coarse woody fuel. Para_NWF, Para_FWF, and Para_CWF denote the fuel-type-specific parameters for NWF, FWF, and CWF, respectively, while Para_PFT1, Para_PFT2, and Para_PFT3 represent the PFT-specific parameters for PFT1, PFT2, and PFT3, respectively.

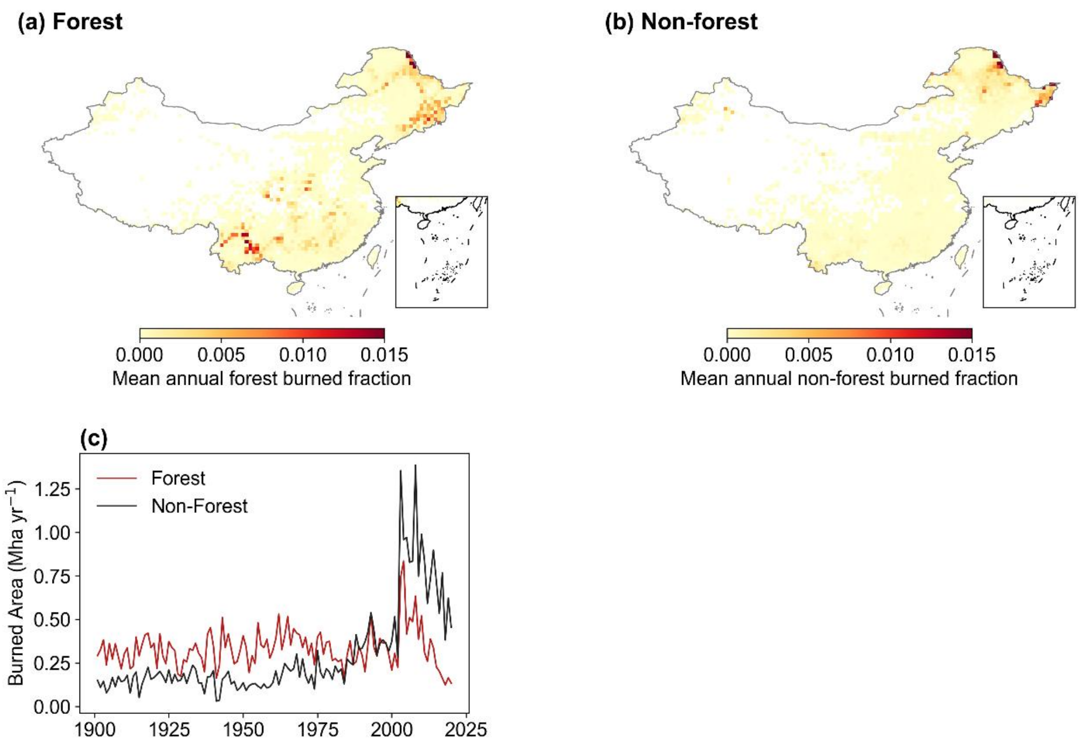

Figure S2. Spatial patterns of burned fraction and temporal variation of burned area in China during 1901–2020, Related to Figures 1 and 2.

(a-b) Spatial distribution of mean annual burned fraction for forest and non-forest at 0.5° × 0.5° resolution.

(c) Interannual variation of total burned area in China.

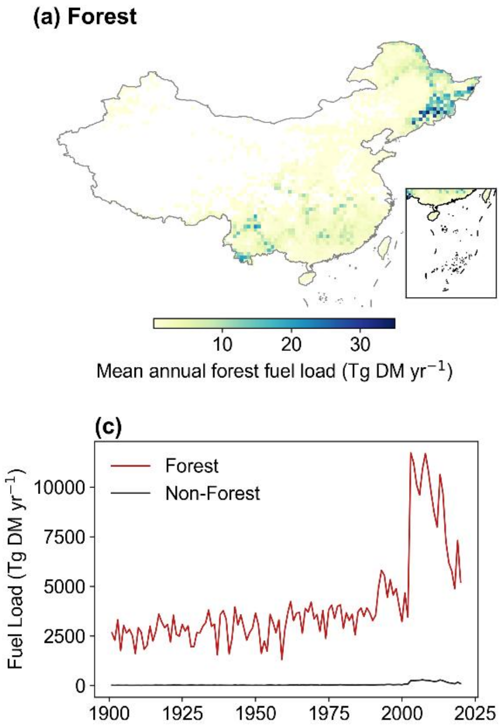

Figure S3. Spatial patterns of fuel load on burned area and its temporal variation in China during 1901–2020, Related to Figures 1 and 2.

(a, b) Spatial distribution of mean annual fuel load on burned area for forest and non-forest at 0.5° × 0.5° resolution.

(c) Interannual variation of total fuel load on burned area in China.

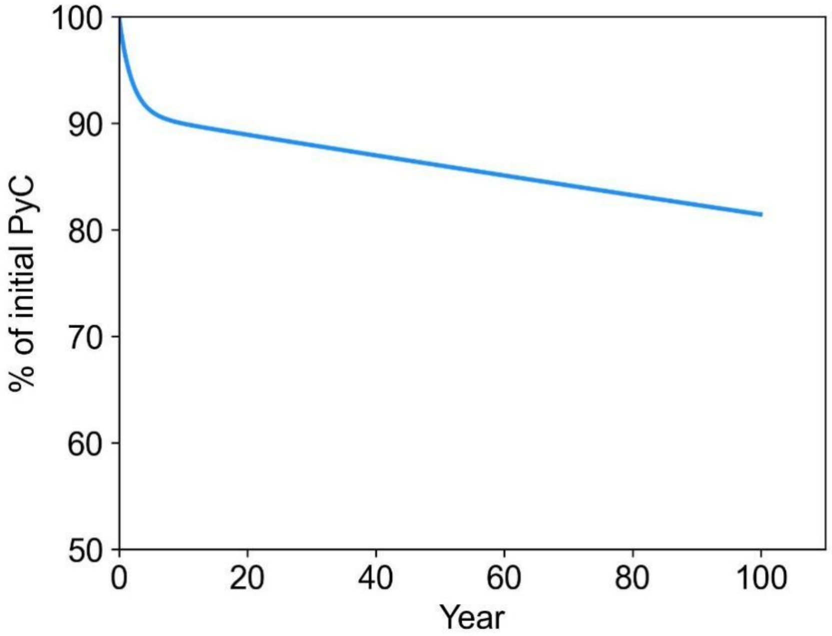

Figure. S4 Decomposition curve of PyC based on a two-pool exponential decay model, Related to Figure 1.

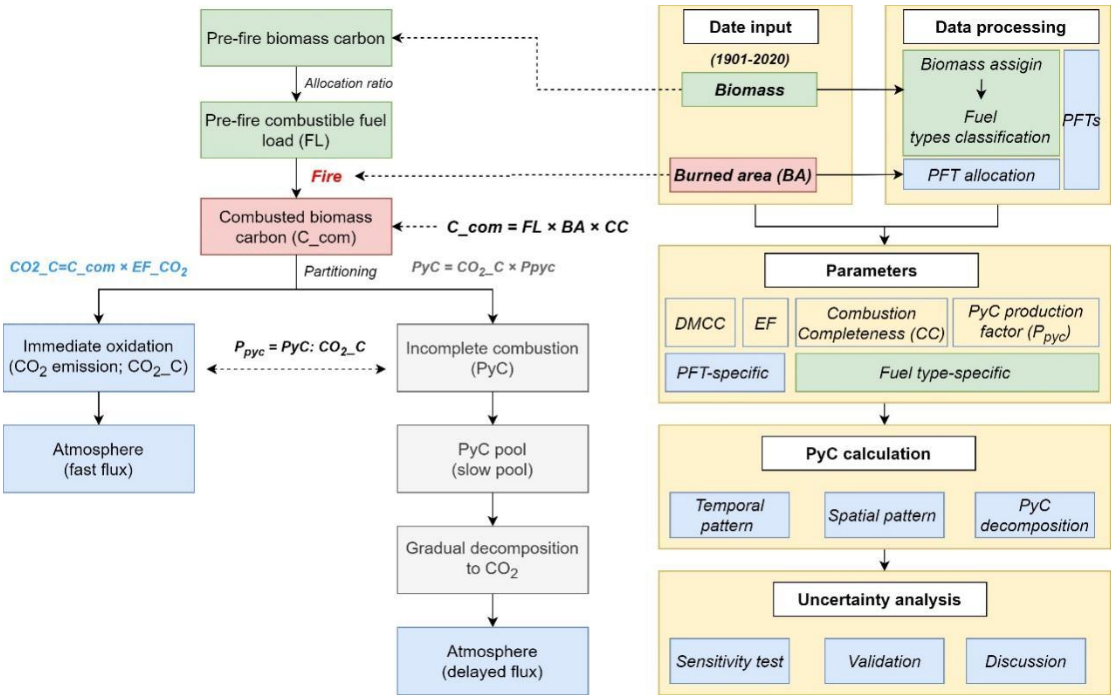

Figure. S5 Conceptual framework illustrating carbon partitioning during biomass burning, Related to Figure 1.

Fire partitions pre-existing biomass carbon into immediate CO_2 emissions and pyrogenic carbon (PyC). While CO_2 is released directly to the atmosphere, PyC enters a longer-lived terrestrial pool and decomposes gradually over time. This process represents a redistribution of carbon residence time rather than a compensatory offset of fire emissions.

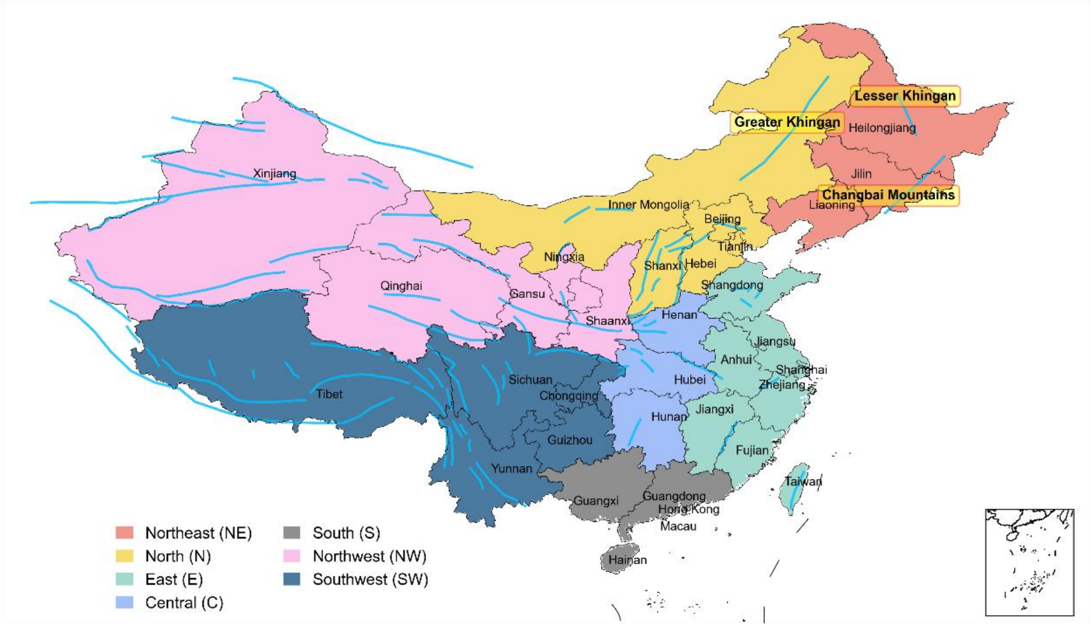

Figure. S6 Region divisions of China used in this study, where blue lines represent major mountain ranges, Related to Figure 2 and Figure 3.

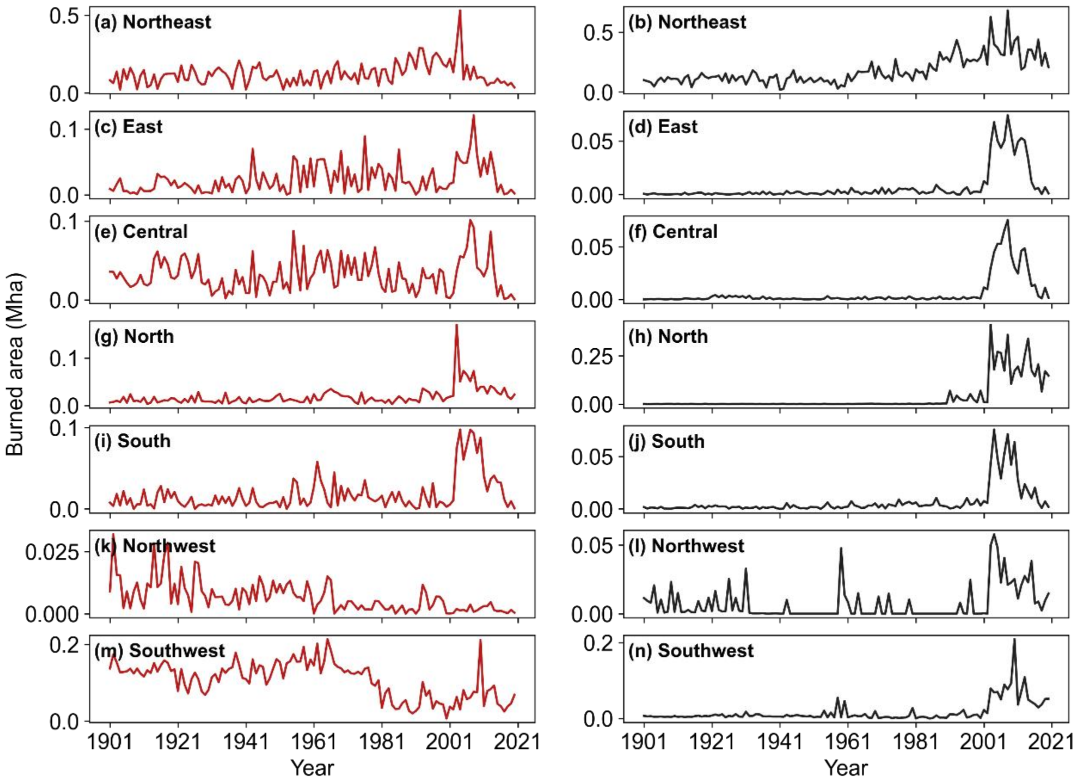

Figure S7. Annual total burned area across seven regions of China from 1901 to 2020, Related to Figure 3.

Panels are arranged by region from top to bottom. The left column shows burned area from forest fires, and the right column shows burned area from non-forest fires.

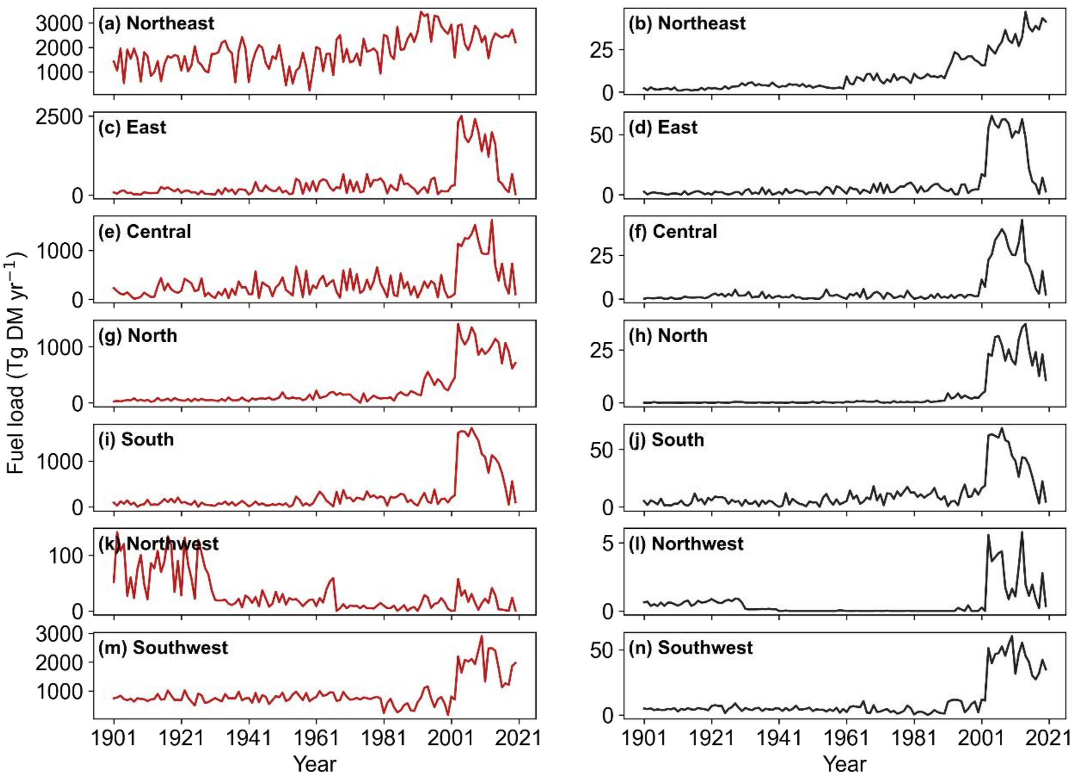

Figure S8. Annual total fuel load on burned area across seven regions of China from 1901 to 2020, Related to Figure 3.

Panels are arranged by region from top to bottom. The left column shows fuel load on burned area from forest fires, and the right column shows fuel load on burned area from non-forest fires.

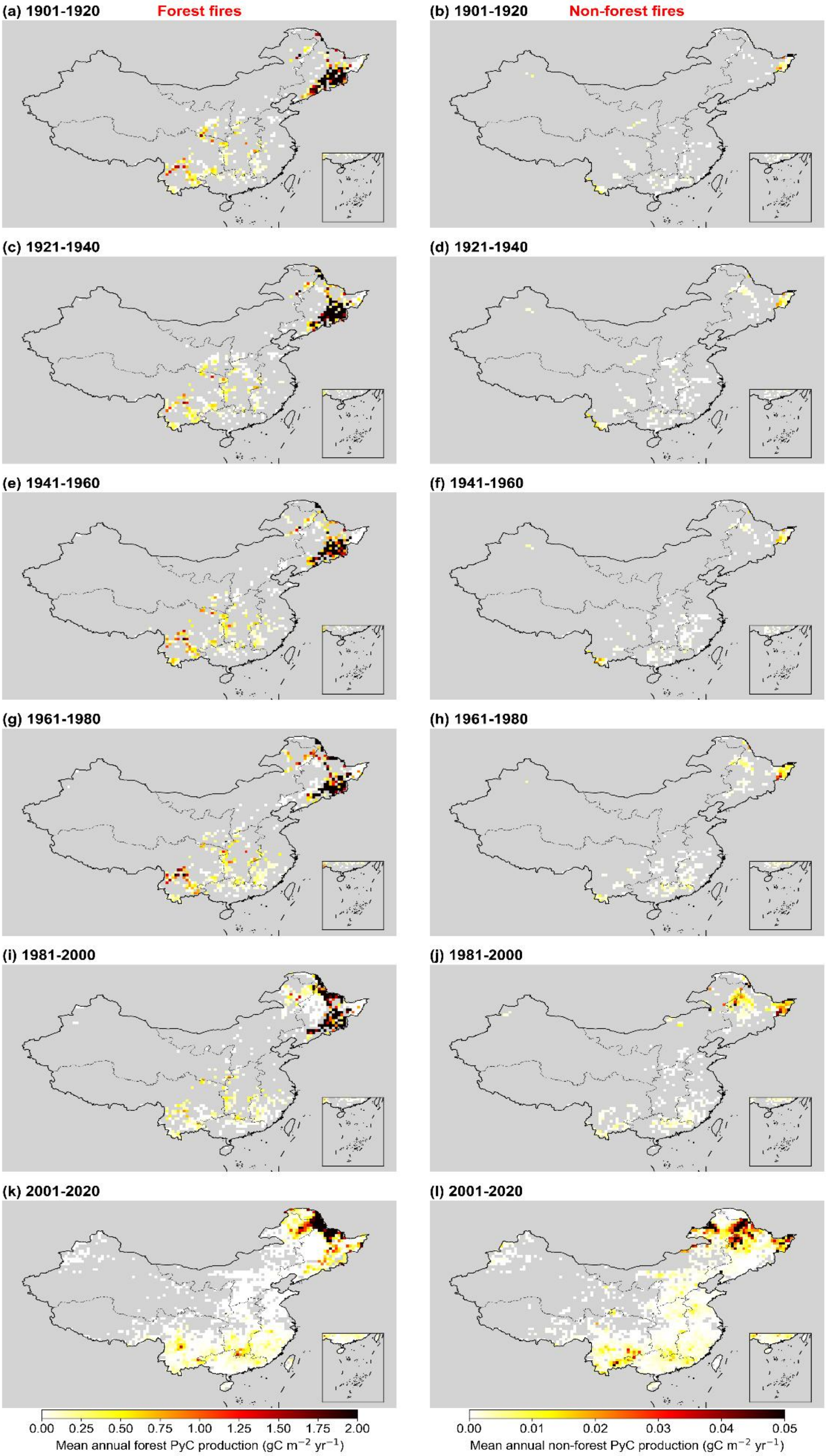

Figure S9. Spatial patterns of PyC production during each 20-year period, Related to Figure 2.

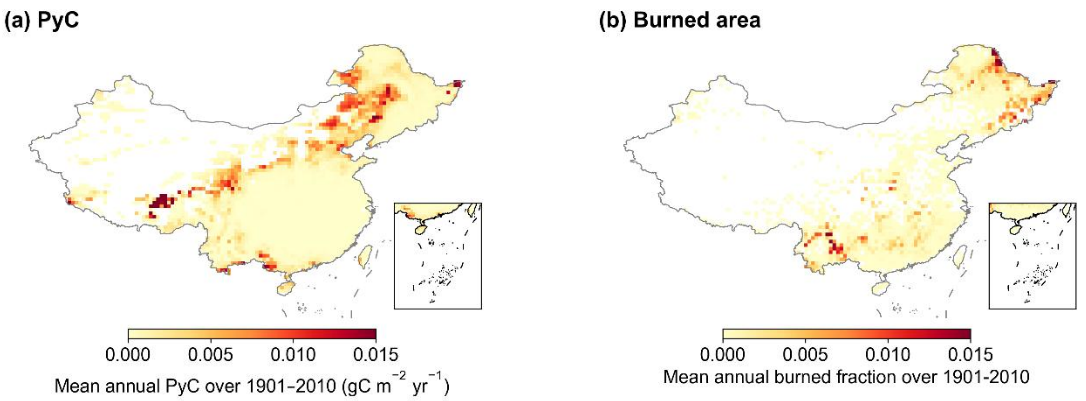

Figure S10. Spatial patterns of mean annual PyC production and burned fraction in China, Related to the Comparison with Other Studies section.

(a) Mean annual PyC production derived from global estimates reported by Bowring et al.[4].

(b) Mean annual burned area fraction over 1901–2010.

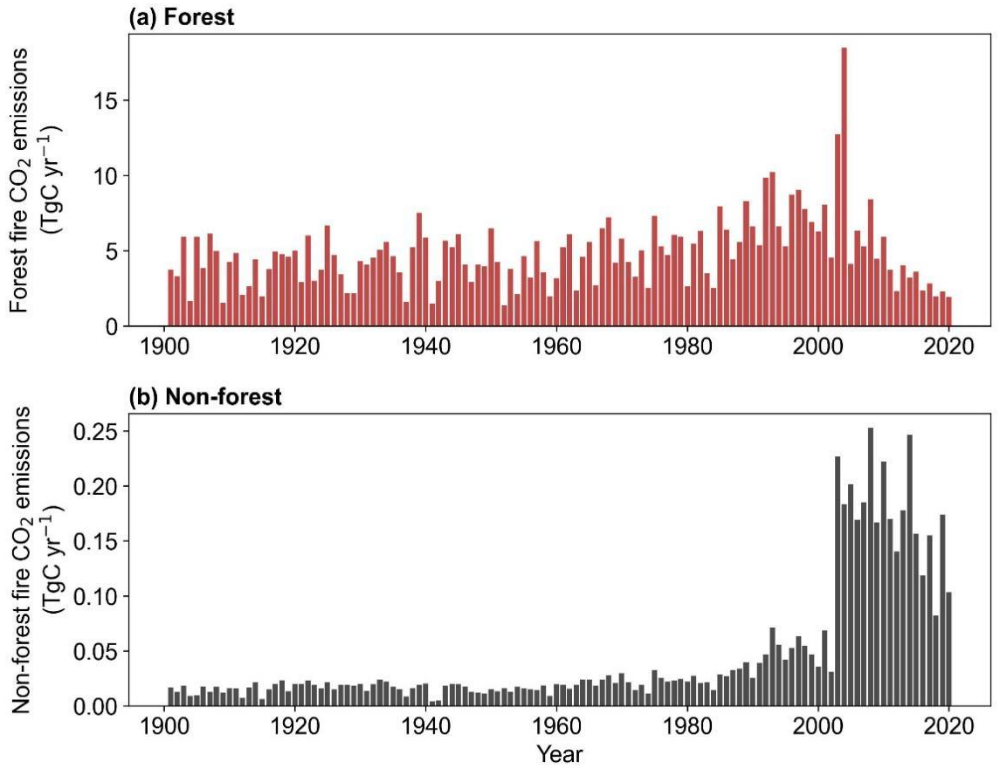

Figure S11. Annual total CO_2 emissions for forest (a) and non-forest (b) fires in China during 1901–2020, Related to Figure 1.

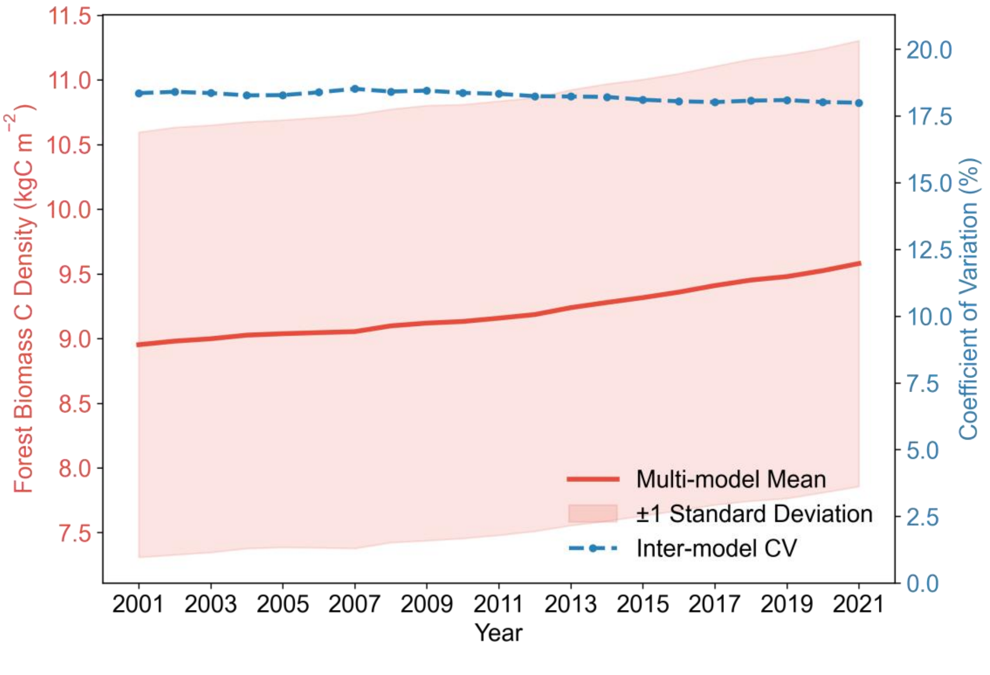

Figure S12. Inter-model comparison of simulated forest biomass carbon density in China (2001–2020) across TRENDY models, Related to Figure 4.

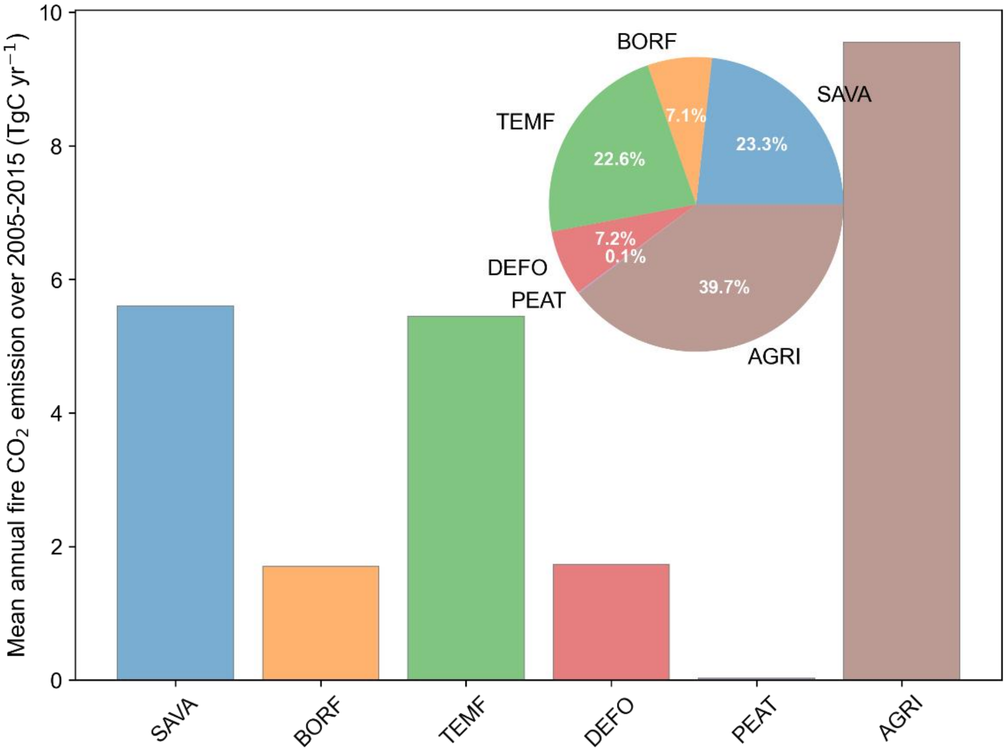

Figure S13. Mean annual fire CO_2 emissions and their relative contributions from different land-cover types in China during 2005-2015, Related to the Uncertainties section.

Fire type includes savanna fires (SAVA), boreal forest fires (BORF), temperate forest fires (TEMF), tropical deforestation fires (DEFO), peatland fires (PEAT), and agricultural fires (AGRI).

Table S1. Parameter values of fire CO_2 emission factors (EF) and dry matter carbon content (DMCC) for different plant functional types (PFTs), Related to the Calculation of PyC production section.

Fire type	No	PFT	DMCC (%)	EF ($gCO_2\ kg^{-1}$ DM)
Forest	1	Tropical broadleaf evergreen	49.18	1643
	2	Tropical broadleaf raingreen	49.18	1643
	3	Temperate needleleaf evergreen	48.94	1647
	4	Temperate broadleaf evergreen	48.94	1647
	5	Temperate broadleaf summergreen	48.94	1647
	6	Boreal needleleaf evergreen	46.50	1489
	7	Boreal broadleaf summergreen	46.50	1489
	8	Boreal needleleaf summergreen	46.50	1489
Non-forest	1	C3 grass	48.83	1686
	2	C4 grass	48.83	1686

Table S2. Parameter values of combustion completeness (CC) and PyC production factor, Related to the Calculation of PyC production section.

Fuel class	Biomass component (ratio)	Fuel class	CC		P_{PyC} (g PyC g^{-1} CO_2-C emitted)		
			Min	Max	95% CI lower	Mean	95% CI upper
1-h fuel	Leaf (100%) + Sapwood (4.5%) + Heartwood (4.5%) + Fruit (100%)	NWF	0.8	1.0	0.074	0.091	0.114
10-h fuel	Sapwood (7.5%) + Heartwood (7.5%)	FWF	0.8	1.0	0.064	0.1	0.153
100-h fuel	Sapwood (21%) + Heartwood (21%)	FWF	0.8	1.0	0.064	0.1	0.153
1000-h fuel	Sapwood (67%) + Heartwood (67%)	CWF	0.2	0.6	0.176	0.261	0.389

Table S3. Key parameters and values used for uncertainty analysis, Related to Figure 4.

Parameter		Meaning	Mean	Standard deviation	Reference
Fuel load (gC m^{-2})		Biomass fuel load	From the ORCHIDEE model	18% variation	Figure S13; Sitch et al.[1]
DMCC (%)	DMCC_TRO	DMCC for tropical forest PFTs	49.18	1.97	van der Werf et al.[2]
	DMCC_TEM	DMCC for temperate forest PFTs	48.94	1.30	
	DMCC_BOR	DMCC for boreal forest PFTs	46.50	3.83	
	DMCC_GRA	DMCC for grassland	48.83	1.27	
EF (gCO_2 kg^{-1} DM)	EF_TRO	EF for tropical forest PFTs	1643	58	van der Werf et al.[2]
	EF_TEM	EF for temperate forest PFTs	1647	37	
	EF_BOR	EF for boreal forest PFTs	1489	121	
	EF_GRA	EF for grassland	1686	38	
P_{PyC} (gPyC g^{-1} CO_2-C)	P_{PyC}_NWF	Ppyc for NWF	0.091	Based on the 95% CI in Table S2	Jones et al.[3]
	P_{PyC}_FWF	Ppyc for FWF	0.100		
	P_{PyC}_CWF	Ppyc for CWF	0.261		

References

1. Sitch, S., O'sullivan, M., Robertson, E., Friedlingstein, P., Albergel, C., Anthoni, P., Arneth, A., Arora, V.K., Bastos, A., and Bastrikov, V. (2024). Trends and drivers of terrestrial sources and sinks of carbon dioxide: An overview of the TRENDY project. Global Biogeochemical Cycles *38*, e2024GB008102.
2. Van Der Werf, G.R., Randerson, J.T., Giglio, L., Van Leeuwen, T.T., Chen, Y., Rogers, B.M., Mu, M., Van Marle, M.J., Morton, D.C., and Collatz, G.J. (2017). Global fire emissions estimates during 1997–2016. Earth System Science Data *9*, 697-720.
3. Jones, M.W., Santín, C., van der Werf, G.R., and Doerr, S.H. (2019). Global fire emissions buffered by the production of pyrogenic carbon. Nature Geoscience *12*, 742-747.
4. Bowring, S.P., Jones, M.W., Ciais, P., Guenet, B., and Abiven, S. (2022). Pyrogenic carbon decomposition critical to resolving fire's role in the Earth system. Nature Geoscience *15*, 135-142.
